# Inhibition and deficiency of the immunoproteasome subunit LMP7 suppress the development and progression of colorectal carcinoma in mice

**DOI:** 10.18632/oncotarget.15141

**Published:** 2017-02-07

**Authors:** Julia Koerner, Thomas Brunner, Marcus Groettrup

**Affiliations:** ^1^ Division of Immunology, Department of Biology, University of Konstanz, Konstanz, Germany; ^2^ Konstanz Research School Chemical Biology (KoRS-CB), University of Konstanz, Konstanz, Germany; ^3^ Biochemical Pharmacology, Department of Biology, University of Konstanz, Konstanz, Germany; ^4^ Biotechnology Institute Thurgau at the University of Konstanz (BITg), Kreuzlingen, Switzerland

**Keywords:** colorectal cancer, immunoproteasome, LMP7, AOM/DSS, Apc^Min/+^

## Abstract

New treatment options and drug targets for colorectal carcinoma are a pressing medical need. Inflammation and pro-inflammatory cytokines produced by Th1 and Th17 cells like IL-6, TNF, IL-17 and IL-23 promote the development and growth of colorectal cancer (CRC). The immunoproteasome is a proteasome subtype highly expressed in immune cells but also in the intestine. Since the immunoproteasome promotes Th1 and Th17 differentiation and pro-inflammatory cytokine production, we investigated here whether deficiency or inhibition of the immunoproteasome subunit LMP7 would interfere with CRC development and exacerbation in preventive and therapeutic mouse models. Treatment with the LMP7 inhibitor ONX 0914 blocked tumor initiation and progression in either chemically-induced (AOM/DSS) or transgenic mouse models (Apc^Min/+^) of colon carcinogenesis. ONX 0914 treatment strongly reduced tumor numbers and CRC-associated loss of body weight while the survival rates were significantly enhanced. Moreover, genetic LMP7 deficiency markedly reduced the tumor burden in AOM/DSS induced wild type and Apc^Min/+^ mice. In conclusion, we show that the immunoproteasome is involved in CRC development and progression and we identify LMP7 as a new potential drug target for the treatment of CRC.

## INTRODUCTION

Colorectal cancer (CRC) is the third most frequently diagnosed and lethal cancer type worldwide [1]. CRC patients are treated with surgical resection, chemotherapy, combined radio-chemotherapy, and recently with monoclonal antibody-based therapies directed to VEGF or EGFR. Nevertheless, every second patient still fails therapy and dies from CRC. CRC develops from adenomatous precursor lesions by a complex interaction of environmental carcinogens, the accumulation of independent genetic aberrations in oncogenes or tumor suppressor genes, and the host immune system [2]. Long-term inflammatory conditions of the gastrointestinal mucosa as a hallmark of patients suffering from inflammatory bowel disease (IBD), including Ulcerative Colitis (UC) and Crohn's Disease (CD), pose a significant risk factor for the development of colon cancer as shown both in clinical studies as well as mouse models of chronic inflammation [3]. CRC increases with severity and duration of intestinal inflammation, demonstrating a tumor-promoting role of inflammation. While the majority of CRC patients have not experienced chronic inflammatory bowel disease before diagnosis, CRCs typically exhibit immune/inflammatory infiltrates referred to as ‘tumor-elicited inflammation’ [4]. The clinical outcome and prognosis of CRC is strongly influenced by the type of immune response generated, especially with respect to T helper cell differentiation (Th1/Th2/Th17/Treg) [5]. For example, CRC tumors with a Th17-dependent transcriptional profile have a poor prognosis [6]. In preclinical mouse models genetic evidence has been provided that cytokines produced by the pro-inflammatory T helper cell subsets Th1 and Th17, in particular tumor necrosis factor (TNF), IL-6, IL-23 and IL-17, promote tumor growth and survival. In Apc^Min/+^ mice, resembling familial adenomatous polyposis, the genetic ablation of IL-17A significantly reduced CRC formation [7]. In inflammation induced mouse models of CRC it was shown that IL-17A deficient mice showed milder colitis, fewer and smaller tumors and expressed reduced levels of IL-6 and TNF [8]. IL-17A and IL-23 cause the attraction of lamina propria myeloid cells (macrophages, granulocytes) to the tumor site which are the major source of IL-6 and TNF. IL-6 and TNF protect premalignant intestinal epithelial cells from cell death and support their proliferation via their signal transducers pSTAT3 and NF-κB, respectively, which induce expression of apoptosis inhibitors (Bcl-XL, survivin) or reduce caspase 9 activation and up-regulate mitogenic factors (PCNA, cyclin D1, cyclin B1, c-Myc) [9, 10]. These insights have nourished the recent proposal that the ablation of pro-inflammatory cytokines might be a valid approach to treat CRC [11].

The constitutive 26S proteasome, a multicatalytic endoprotease, is responsible for the degradation of regulatory as well as misfolded intracellular proteins, thereby generating 8-10 residue peptides for presentation on MHC class I molecules to CD8^+^ T lymphocytes [12]. The proteolytically active sites are located within the internal chamber of the 20S proteasome which consists of 28 subunits arranged into four stacked rings [13]. The central two rings are each composed of seven distinct β-subunits, three of which (β1, β2, and β5) exhibit the catalytically active sites of the constitutive 20S proteasome. Stimulation of cells with pro-inflammatory cytokines such as IFN-γ or TNF leads to replacement of the three constitutive catalytically active proteasome subunits by the inducible active β subunits low molecular mass peptide 2 (LMP2, β1i), multicatalytic endopeptidase complex-like (MECL)-1 (β2i), and LMP7 (β5i). These subunits are incorporated into the 20S core particle, thereby forming the immunoproteasome, which is expressed in inflamed tissue as well as constitutively in immune cells [14]. The immunoproteasome shapes the antigenic repertoire of MHC class I-restricted T cell epitopes at sites of inflammation resulting in more efficient activation of cytotoxic T Lymphocytes (CTLs) [15, 16]. Apart from antigen processing, the immunoproteasome was recently shown to have additional functions such as influencing cytokine production and T helper cell differentiation, as well as T cell survival. This led to the hypothesis that subunit-selective inhibition of the immunoproteasome may be an ideal approach to attenuate undesired T cell responses in inflamed tissue. Inhibition of the β5i subunit of the immunoproteasome by the cell-permeable, irreversible epoxyketone inhibitor ONX 0914 (formerly PR-957) was shown to modulate cytokine production and to block presentation of LMP7-specific MHC-I restricted antigens [17], thus lowering T cell activation as well as proliferation. Immunoproteasome inhibition ameliorated clinical symptoms in several mouse models of autoimmune diseases *in vivo*, including rheumatoid arthritis [14], type 1 diabetes, systemic lupus erythematosus (SLE) [18], Hashimoto's thyroiditis [19], and multiple sclerosis [20]. Additionally, it was shown that ONX 0914 treatment suppressed the development and differentiation of pro-inflammatory Th1 and Th17 responses while enhancing the generation of anti-inflammatory regulatory T cell (Tregs) *in vitro* [21]. Two previous studies revealed that inhibition of the chymotrypsin-like activity of LMP7 with ONX 0914 as well as genetic deletion of LMP7 reduced the pathology in dextran sodium sulfate (DSS) induced experimental colitis. ONX 0914 treatment resulted in suppressed pro-inflammatory cytokine production, less inflammation and tissue destruction, as well as reduced differentiation of CD4^+^ T cells into Th17 cells [21–23]. These findings suggest ONX 0914 treatment as a promising therapeutic intervention with colitis-associated pathologies. Furthermore the observed suppressive effects of LMP7 inhibition on the production of pro-inflammatory cytokines indicate a potential treatment option against inflammation-associated colon cancer. Previous reports established elevated expression of LMP2 and LMP7 in approximately 70% of human CRC tumor samples [24, 25] which might facilitate direct targeting of malignant CRC cells by ONX 0914. Thus, ONX 0914 could potentially interfere with inflammation-driven CRC development during the preceding inflammation phase or directly with the growth of already established tumors. In this study we could indeed show that ONX 0914 suppressed the emergence and progression of CRC in preclinical mouse models in preventive and therapeutic settings.

## RESULTS

### Treatment with the LMP7 inhibitor ONX 0914 suppresses the formation of inflammation-mediated colon carcinogenesis in the AOM/DSS model

**Figure 1:**
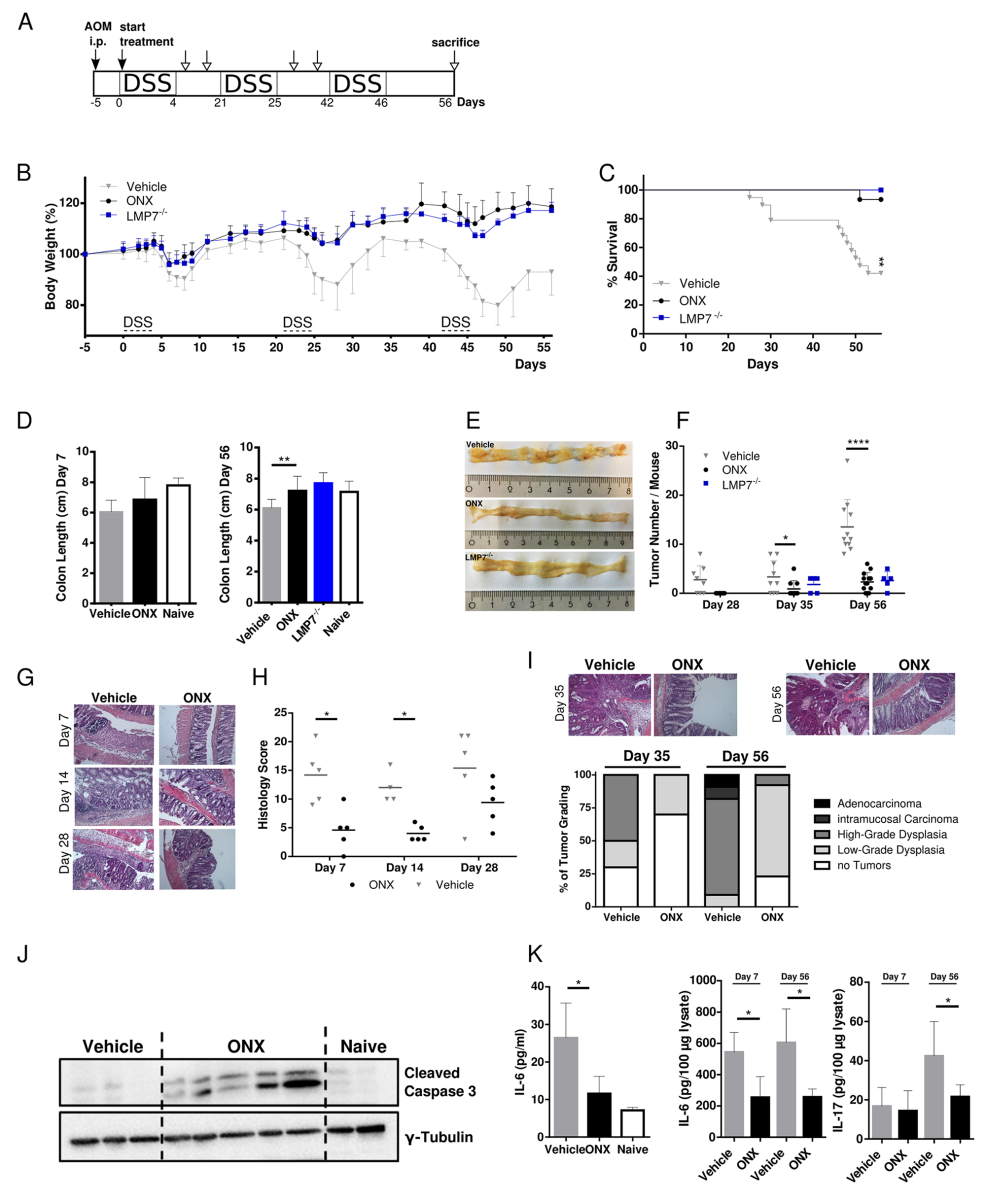
Treatment with ONX 0914 suppresses AOM/DSS-induced colorectal tumor formation **A.** Schedule of the AOM/DSS mouse model. **B.** CAC was induced in 8-week old C57BL/6 or LMP7^-/-^ mice by intraperitoneal injection of AOM at 12 mg/kg and subsequent oral administration of 2 % DSS in drinking water for 5 days in 3 cycles which was interrupted by 2-week periods without DSS challenge. Mice were treated with vehicle (grey triangles) or 10 mg/kg ONX 0914 (black dots) s.c. every second day from day zero of the first DSS cycle. LMP7^-/-^ mice (blue squares) were not inhibitor treated (n ≥ 5). Body weight (bw) was recorded daily during DSS cycles and every second day after recovery. Data are presented as percent loss of bw compared to the initial bw on day 5. Data are means ± SEM. i.p. intraperitoneal. **C.** Survival curve showing the percentage of mice remaining alive until the end of the experiment on day 56. Mice were sacrificed due to disease symptoms such as extensive loss of bw, obstipation, rectal bleeding, rectal prolapse or moribund health condition. **D.** Average colon length of vehicle (grey columns) or ONX 0914 (black columns) treated wild type mice and LMP7^-/-^ mice (blue column) sacrificed at day 7 (left panel) and at the endpoint of the experiment on day 56 (right panel). Naïve C57BL/6 mice were used as an untreated control (white columns). **E.** On day 7, 14, 28, 35 and 56 colons were removed and analyzed macroscopically for tumor lesions. Representative gross pathology of the descending colon and rectum at the end of the AOM/DSS protocol on day 56. **F.** Graph shows number of tumor lesions in the large intestine by semi quantitative macroscopic assessment. Tumors are defined as lesion ≥ 2 mm. Data are means ± SEM. **G.** Representative images of H&E stained paraffin-embedded colon sections. **H.** Histology score of colon sections was defined by colitis scoring at day 7, 14 and 28. In brief, intestinal inflammation was defined on the basis of 4 different parameters: Inflammation severity, extent of inflammation, crypt damage and involvement. **I.** H&E stainings of colon sections of vehicle or ONX 0914 treated mice on day 35 (upper panel, left) and day 56 (upper panel, right) were quantified according to the grading of the respective tumor types (lower panel) that are low-grade dysplasia (light grey), high-grade dysplasia (grey), intramucosal carcinoma (dark grey) or adenocarcinoma (black). The percentage of mice having no tumors are represented by white bars. **J.** Western blot analysis of cleaved caspase 3 in whole colon tissue lysates (one mouse per lane) on day 56. γ-tubulin was used as loading control. **K.** IL-6 levels in blood serum (left panel) and IL-6 and IL-17A cytokine levels of day 7 and day 56 in whole-colon lysates (right panel) of vehicle (grey columns) and ONX 0914 (black columns) treated AOM/DSS mice. Data are means ± SEM. Serum of naïve C57BL/6 mice was used as control (white columns). Data are representative of 3 independent experiments. *p < 0.05, **p < 0.005, ****p < 0.00005.

Previously it has been shown that LMP7 deficiency and inhibition suppressed experimental colitis after induction with DSS [22, 23] as well as in T cell transfer colitis [21]. In order to investigate the consequence of LMP7 inhibition in the setting of inflammation-associated colon tumorigenesis, we used the two-step AOM/DSS model, which is a well-established mouse model of colitis-associated carcinogenesis (CAC). This model is suitable to study the contribution of the tumor microenvironment, as it combines a chemical pro-carcinogen in the context of chronic inflammation to recapitulate tumor initiation and progression in a well-defined chronology [26]. C57BL/6 mice were treated with a single dose of the DNA-alkylating agent azoxymethane (AOM), which specifically induces adenomas in the distal colon. The formation of colitis-associated colonic tumors is accelerated by oral application of DSS which vulnerates the gut mucosa thus inducing release of luminal gut microbiota and consequently long-term chronic intestinal inflammation (Figure 1A).

ONX 0914 treatment started together with the first DSS cycle and mice were then treated with the inhibitor or vehicle control every other day for 8 weeks in total. We compared the clinical outcome in vehicle and ONX 0914 treated mice by comparing loss of body weight as a characteristic symptom of DSS mediated intestinal inflammation. Even after the first DSS cycle differences in body weight loss were detectable, as ONX 0914 treated mice were protected from losing weight in the acute colitis phase with improved recovery to initial body weight. Similar to ONX 0914 treated wild type mice, LMP7^-/-^ mice also develop only mild symptoms of the disease and were almost fully protected from weight loss during all cycles of DSS. This difference increased with the following DSS cycles during the course of the experiment (Figure 1B). Vehicle treated mice lost body weight by about 25%, especially after the last cycle of DSS, resulting in the need of sacrificing these mice due to induced chronic colitis accompanied with bloody stool and the occurrence of rectal prolapse. Additionally, treatment with the inhibitor shows extended survival compared to vehicle-treated mice (Figure 1C). Notably, 60% of the animals in vehicle treated groups died or had to be sacrificed before termination of the experiment even at early (day 22) as well as at late stages, whereas only one mouse of the ONX 0914 treated animals died during the course of 3 independent experiments. LMP7 knockout (KO) mice as well showed an increased lifespan in the AOM/DSS model. Consistent with the major weight loss, vehicle treated mice exhibited a tendency for shortening of colons in the acute phase (day 7, Figure 1D, left panel), as well as significantly shortened colons at the endpoint of the experiment probably due to severe pathologic inflammation and increased fibrosis in colonic mucosal tissue at later stages (day 56, Figure 1D, right panel). In contrast, inhibitor-treated mice, as well as LMP7 KO mice displayed colon lengths similar to naïve untreated mice. At the endpoint of the experiment on day 56 we observed tumors in the distal and mid colon with rare occasional lesions in the proximal colon (Figure 1E). Notably, treatment with ONX 0914 attenuated or even suppressed tumor growth, whereas vehicle control mice exhibited a significant increase in tumor nodule number (Figure 1F). In addition, average tumor sizes tended to be enlarged in vehicle treated mice compared to the two other experimental groups (graph not shown). LMP7^-/-^ mice blocked tumor multiplicity and dysplastic lesions similarly to the effect of ONX 0914 treatment in C57BL/6 mice. Additionally, inhibitor treated mice exhibited lower levels of inflammation as indicated by decreased histology scores (Figure 1G and 1H). Histological examination of acute and chronic phases of inflammation on day 7, day 14, and day 28 showed that vehicle treated mice presented with destruction of the intestinal architecture with almost complete crypt loss and massive leukocyte infiltration into submucosal layers. Conversely, treatment with the inhibitor only lead to moderate signs of inflammation, with restored crypt architecture and reduced inflammation in the lamina propria (Figure 1G). Although, histology scores were not completely reduced to levels of naïve mice, ONX 0914 treatment limited tissue damage and suppressed the ensuing inflammatory response which contributes to reduced tumor burden and disease pathology.

In accordance with the macroscopically visible significant decrease in tumor multiplicity, histopathological quantification of intestinal adenomas supported reduction of gross tumor number in ONX 0914 treated mice, as illustrated in Figure 1I. Mice treated with the LMP7 inhibitor developed lower incidences of dysplastic regions compared to vehicle control. The majority of lesions were characterized as mild hyperplasia and low-grade adenomas with rarely occasional appearance of carcinoma *in situ*, displaying lesions in a less advanced stage with mostly focal, small sized residual colitic lesions. Vehicle treated mice show a prevalence of high-grade dysplasia with frequent invasion into the submucosa. Evidence of adenocarcinoma as characterized by published pathohistological scoring systems [27] was only observed in vehicle-treated mice. The occurrence of apoptosis was confirmed via western blotting for cleaved caspase 3. Figure 1J revealed significant differences in the presence of activated caspases in tumor adjacent areas compared to the absence of active caspases in vehicle treated or naïve B6 colons, suggesting the prevention of CAC during chronic inflammation through pro-apoptotic effects by treatment with the inhibitor.

Furthermore, we analyzed the expression of pro-inflammatory cytokines in colon tissue and serum of diseased mice. As previously described, IL-6 is the key cytokine to be elevated in CAC and CRC patients, thus playing an important role in development of mucosal inflammation associated with colon cancer [8]. We therefore assessed the level of IL-6 in serum by ELISA. As shown in Figure 1K (left panel), LMP7 inhibition led to significantly decreased serum IL-6 levels as compared to control mice. In addition, we analyzed the content of IL-6 and IL-17A in lysates of colon tissue. IL-6 cytokine levels were reduced in mice treated with the inhibitor in the acute inflammatory phase (day 7), as well as at the endpoint of the experiment (day 56), which underlines the observed impact of ONX 0914 on the clinical outcome in AOM/DSS treated mice most likely by limiting inflammation-mediated tumor development in the colons of AOM/DSS mice. There was no significant difference observed for IL-17A content in colon lysates on day 7. However, cytokine levels were reduced in ONX 0914 treated mice on day 56 (Figure 1K, right panel). Collectively, this data show that selective inhibition of the immunoproteasome effectively protects against tumor initiation and progression *in vivo* during acute phases of intestinal inflammation, as well as in subsequent stages of tumor development.

### Treatment with the LMP7 inhibitor ONX 0914 suppresses colorectal tumor growth in the absence of acute inflammation in a therapeutic setting

**Figure 2:**
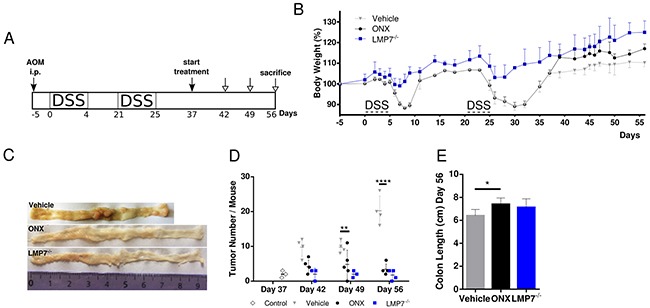
Treatment with ONX 0914 suppresses growth of already established tumors in a therapeutic setting after cessation of inflammation induction **A.** Scheme of the experimental procedure. **B.** CAC was induced in 8-week old C57BL/6 or LMP7^-/-^ mice by i.p. injection of AOM at 12 mg/kg and subsequent oral administration of 2 % DSS in drinking water for 5 days in only 2 cycles (day 0-4, day 21-25). DSS cycles were interrupted by 2-week periods applying tap water. Mice were treated with vehicle (grey triangles) or 10 mg/kg ONX 0914 (black dots) s.c. every second day from day 37 onwards, when mice gained their original weight after the second DSS cycle. LMP7^-/-^ mice (blue squares) were not inhibitor treated (n ≥ 4). The body weight (bw) was recorded daily during DSS cycles and every second day thereafter. Data are presented as percent loss of bw compared to initial bw on day -5. Data are means ± SEM. **C.** On day 37, 42, 49 and 56 colons were removed and analyzed macroscopically for the presence of tumor lesions. Representative gross pathology of the descending colon and rectum at the end of the AOM/DSS protocol on day 56 is shown. **D.** The graph shows the numbers of tumor lesions in the large intestine of vehicle or ONX 0914 treated mice and LMP7^-/-^ mice by semi quantitative macroscopic assessment. Additionally, AOM/DSS control mice (white diamonds) were analyzed on day 37 for tumor incidence. Tumors are defined as lesion ≥ 2 mm. Data are means ± SEM. **E.** Average colon length on day 56 of vehicle or ONX 0914 treated mice and LMP7^-/-^ mice. *p < 0.05, **p < 0.005, ***p < 0.0005, ****p < 0.00005.

Our study of ONX treatment using the AOM/DSS model of CRC suggests that the immunoproteasome is involved in initiation and progression of CAC, where inflammation plays a major contributing role to cancer development. Next, we addressed immunoproteasome inhibition as a therapeutic approach after tumors had emerged and in the absence of acute DSS-induced inflammation. To this aim, we adapted the AOM/DSS model such that the third DSS cycle was omitted and started treatment only after mice had regained their original weight, which correlates to cessation of the acute inflammatory phase (Figure 2A). Untreated control mice were bearing already established tumors on day 37 (Figure 2D) when we started ONX 0914 treatment every other day until the endpoint of the AOM/DSS model on day 56. Already after a single application of the inhibitor, we observed a profound increase in body weight of mice treated with ONX 0914, whereas mice only treated with vehicle stayed at a constantly low body weight level after recovery from DSS-induced inflammation (Figure 2B). Intriguingly, treatment with ONX 0914 halted further progression of tumor growth, since tumor number arrested at the same level for the next three time-points analyzed (day 42, day 49 and day 56; Figure 2D) as compared to tumor-bearing control mice. More interestingly, LMP7-deficient mice, which were as well protected from body weight loss during the two precedent DSS cycles, showed similar tumor number as in the ONX 0914 treated animals (Figure 2C and 2D). In addition to alleviation of the loss of body weight and reduction of total tumor numbers, ONX 0914 treatment suppressed colon shortening as seen in vehicle treated mice at the end of the experiment (Figure 2E). Taken together, these data suggest that ONX 0914 treatment curtails tumor progression in the absence of exogenous acute inflammatory stimuli by counteracting further expansion of adenomas that already had formed in this model but also by suppression of new colonic lesions.

### ONX 0914 treatment reduces tumorigenesis in the Apc^Min/+^ mouse model

Compared to the chemically induced and inflammation-associated AOM/DSS model, which is characterized by activating mutations in epithelial pathways such as p53 and K-Ras [26, 28], the Apc^Min/+^ mouse model relies on deregulation of the Wnt/β-catenin signaling cascade in the first place, therefore addressing another route of intestinal carcinogenesis development. Transgenic Apc^Min/+^ mice carry an ENU-induced germline missense mutation in one allele of the APC (adenomatous polyposis coli) tumor suppressor gene which results in truncation of the Apc protein. Somatic loss of the remaining wild-type allele leads to accumulation and nuclear translocation of β-catenin aberrantly activating Wnt signaling pathway, which subsequently drives neoplastic transformation within the crypt axis and subsequently resulting in the development of multiple polyps throughout the whole intestinal tract [29]. The Wnt-driven Apc^Min/+^ mouse model quickly recapitulates both genetic, as well as inflammatory processes of human sporadic intestinal tumorigenesis by exhibiting polyposis following loss of APC within 16 to 20 weeks of age [30, 31]. Homologous to human *Apc*, deregulation of Wnt/β-catenin signaling following inactivating mutation in the wild-type allele of this gene is the major initiating factor in the etiology and pathogenesis of familial adenomatous polyposis (FAP) and frequently found in more than 80% of sporadic CRC in human patients [30, 32]. Apc^Min/+^ mice and FAP patients share several phenotypic characteristics in manifestation of lesions. However, whereas most of the multiple intestinal tumors occur in the small intestine in the Apc^Min/+^ mouse, tumors in humans are predominantly localized in the colon.

**Figure 3:**
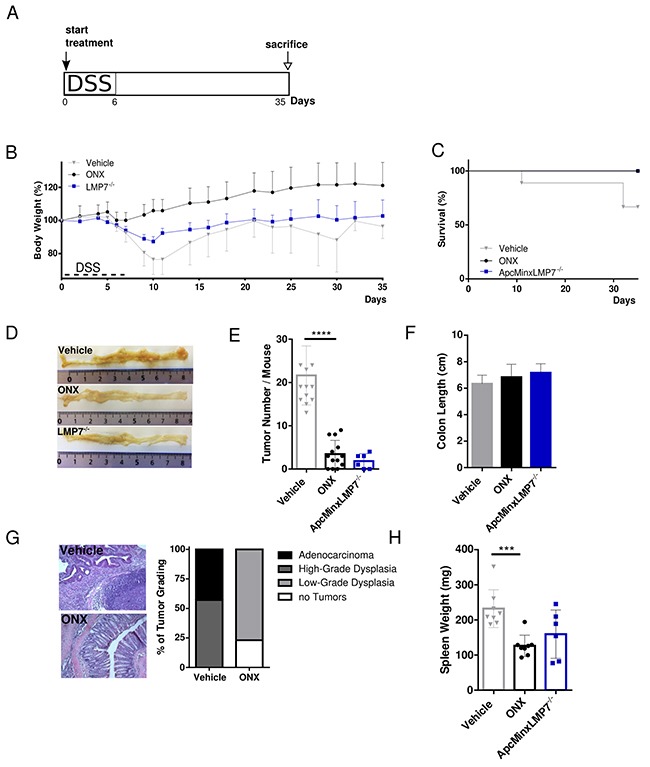
Treatment with the LMP7 inhibitor ONX 0914 suppresses intestinal tumorigenesis in Apc^Min/+^ mice **A.** Scheme of experimental procedures: Induction of colon tumorigenesis was accelerated by oral administration of 2 % DSS for 7 consecutive days. Five-week old Apc^Min/+^ or Apc^Min/+^LMP7^-/-^ mice were treated every other day with either vehicle or 10 mg/kg ONX 0914 s.c. from day 0 of the DSS cycle until the endpoint of the Apc^Min^/DSS protocol on day 35 (n ≥ 5). **B.** Body weight of vehicle (grey triangles) or inhibitor (black dots) treated Apc^Min/+^ mice and Apc^Min/+^LMP7^-/-^ mice (blue squares) was recorded daily during the DSS cycle and every other day after recovering to original weight. Data are illustrated as percent weight loss (y-axis) plotted versus time (x-axis). Data points represent means ± SEM. **C.** Survival curve showing the percentage of vehicle or ONX 0914 treated Apc^Min/+^ mice and Apc^Min/+^LMP7^-/-^ mice remaining alive until the end of the experiment on day 35. **D.** On day 20 or 35 after beginning of the experiment, colons were removed and opened longitudinally for semi quantitative macroscopic analysis of colonic tumor formation. Pictures are representative macroscopic views of colons on day 35. **E.** Graph shows tumor number per mouse as defined by lesions ≥ 2 mm. Data are means ± SEM. **F.** Average colon length on day 35 of vehicle (grey column) or ONX 0914 (black column) treated Apc^Min/+^ and untreated Apc^Min/+^/LMP7^-/-^ mice (blue column). **G.** H&E stainings of colon sections of vehicle or ONX 0914 treated Apc^Min/+^ mice on day 35 were quantified according to the grading of the respective tumor types that are low-grade dysplasia (light grey), high-grade dysplasia (grey) or adenocarcinoma (black). The percentage of mice having no tumors are represented by white bars. **H.** Individual spleen weight on treatment day 35 of vehicle or ONX 0914 treated Apc^Min/+^ and Apc^Min/+^LMP7^-/-^ mice. Graph represents data as means ± SEM. Data are compiled from two independent experiments. ***p < 0.0005, ****p < 0.00005.

We started ONX 0914 treatment in Apc^Min/+^ mice at 5 weeks of age, as it is known that multiple intestinal polyposis spontaneously manifest early in life [33]. However, at this age no macroscopically visible tumor nodules were observed (own experience, unpublished). In addition, LMP7 KO mice were crossed onto Apc^Min/+^ background in order to investigate the influence of genetic LMP7 deficiency on intestinal tumorigenesis in the Apc^Min/+^ model. Either female or male mice were randomly distributed into vehicle or ONX 0914 treated groups; Apc^Min/+^LMP7^-/-^ mice were left untreated. These mice were initially given a one-week cycle of DSS which selectively promotes the development of colonic neoplasms [34] (Figure 3A). ONX 0914 treatment started from day 0 of the experiment with continuous treatment every other day until the endpoint of the experiment on day 35.

The profound weight loss of vehicle-treated mice after the DSS cycle was mostly prevented in Apc^Min/+^ mice treated with the LMP7 inhibitor. In addition, mice of the vehicle group did not recover to original weight after regeneration post DSS treatment and stayed at a constantly low body weight level and lost weight again gradually from day 25 onwards until death or the endpoint of the experiment on day 35 (Figure 3B). In contrast to the consequence of LMP7 knock out in the AOM/DSS model, we observed a decrease in total body weight in Apc^Min/+^ LMP7^-/-^ mice, however not as severe as seen in Apc^Min/+^ mice of the vehicle group. ONX 0914 treatment, as well as genetic deletion of the LMP7 subunit in Apc^Min/+^ mice lead to increased overall survival, whereas animals which received only vehicle died or had to be sacrificed (Figure 3C). These mice probably succumb to consequence of secondary effects due to tumor growth as a result of severe intestinal obstruction. Noticeably, macroscopic examination of colons at the endpoint of the experiment on day 35 showed that ONX 0914 treated mice, as well as the Apc^Min/+^ LMP7^-/-^ mice developed none or decreased colorectal tumor incidences compared to vehicle treated mice in a highly significant manner (Figure 3D and 3E). In comparison, vehicle treated Apc^Min/+^ mice show clear evidence of occult bleeding throughout colonic and rectal segments with extensive polyposis and multiple large tumors in the colon (Figure 3D). Additionally, ONX 0914 treatment also had an effect on tumor size, suggesting that ONX 0914 suppressed tumor outgrowth, as well as tumor initiation. Moreover, the loss of LMP7 profoundly decreased the incidence and multiplicity of colonic adenomas in Apc^Min/+^LMP7^-/-^ mice, as well. Furthermore, colons of vehicle treated mice tended to differ in their length, i.e. we found slightly reduced colon lengths at the endpoint of the experiment compared to ONX 0914 treated or LMP7 KO control Apc^Min/+^ mice (Figure 3F). Histomorphologic analysis of the large intestine confirmed the macroscopically visible phenotype of reduced tumor numbers in ONX 0914 treated mice. Colon sections of these mice showed mostly preserved intestinal architecture, albeit with lymphocyte infiltration as well as enlarged crypts. On the contrary, colon sections of vehicle treated mice showed severe intestinal inflammation and high-grad hyperplasia characterized by crypt damage, necrosis of the epithelial layer, large follicles of lymphocyte infiltration, the loss of goblet cells and poorly differentiated lesions with hyperchromatic cells extending into the submucosa (Figure 3G).

As mice suffer from anemia, this commonly initiates splenomegaly formation due to splenic hematopoiesis in the context of disease progression [35]. Thus, we measured spleen weight and size at sacrifice and found that vehicle treated mice had significantly enlarged spleens compared to mice treated with the LMP7 inhibitor. Spleen weights of Apc^Min/+^LMP7^-/-^ mice were similar to those of Apc^Min/+^ mice treated with ONX 0914 (Figure 3H). Despite the phenotype of splenomegaly, no metastases to lymph nodes, liver or lungs have been detected. Strikingly, these data show that LMP7 inhibition as well as genetic deletion of this immunoproteasome subunit significantly suppresses intestinal polyp development in the Apc^Min/+^ mouse model of sporadic intestinal tumorigenesis.

### Inhibition of the immunoproteasome is also efficacious in therapeutic treatment of already established tumors in the Apc^Min/+^ mouse model

**Figure 4:**
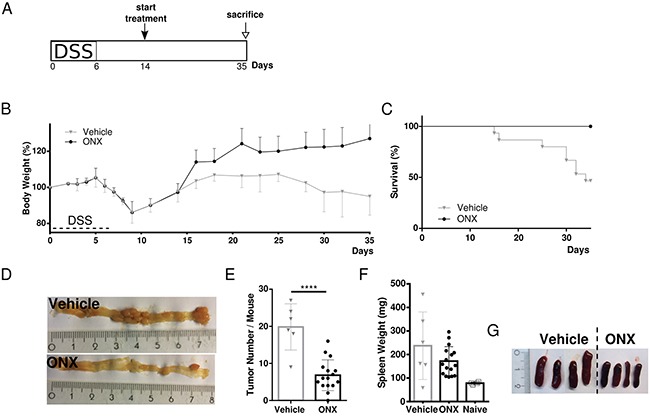
Therapeutic treatment with the LMP7 inhibitor ONX 0914 suppresses already established tumors in Apc^Min/+^ mice **A.** Scheme of induction of colon tumorigenesis in Apc^Min/+^ mice as accelerated by oral administration of 2 % DSS for 7 consecutive days. **B.** Five-week old Apc^Min/+^ mice were treated every other day with either vehicle or 10 mg/kg ONX 0914 s.c. starting from day 14 (n ≥ 5). The body weight of individual mice was recorded daily during the DSS cycle or every other day after recovering to original weight. Data are illustrated as percent weight loss of the initial bw at day 0 of the experiment. Data points represent means ± SEM. **C.** Survival curve showing the percentage of mice remaining alive until the end of the experiment on day 35. **D.** On day 35 colons were removed and opened longitudinally for macroscopic assessment of tumor formation. Pictures are representative macroscopic views of colons on day 35. **E.** Colon tumor numbers of vehicle (grey triangles) or ONX 0914 (black dots) treated Apc^Min/+^ scored on day 35 as defined by lesions ≥ 2 mm are displayed. Data are means ± SEM. **F.** Individual spleen weights of vehicle (grey triangles) or ONX 0914 (black dots) treated Apc^Min/+^ on day 35 of treatment. Graph represents data as means ± SEM. Spleens of naïve 6-week old Apc^Min/+^ mice were used as a control (white squares). **G.** Representative images of individual spleen sizes of vehicle or ONX 0914 treated Apc^Min/+^ on day 35. Data have been compiled from two independent experiments. ****p<0.00005.

In order to further assess anti-tumorigenic efficacy of ONX 0914 treatment in already established tumors, we analyzed the effect of immunoproteasome inhibition in a therapeutic setting of the Apc^Min/+^ mouse model. In order to accelerate tumor formation specifically in colonic segment, we made again use of the Apc^Min/+^-DSS mouse model. Treatment with either vehicle or the LMP7 inhibitor started after mice had regained their original weight on day 14. The inhibitor was then applied every other day until the end of the experiment on day 35 (Figure 4A). Once more, we observed rapid increase in body weight after the first injection of the inhibitor. After that, body weights of ONX 0914 treated Apc^Min/+^ mice steadily increased. Compared to that, vehicle treated Apc^Min/+^ mice started losing weight at day 7 and did not recover to initial body weight with even decreasing body weight levels in the last days of the experiment (Figure 4B). Remarkably, animals which received only vehicle showed significantly reduced survival (Figure 4C). Half of the mice succumbed to tumor complications, as seen by the severe phenotype accompanying disease progression with intestinal occlusion, excessive weight loss or rectal prolapse. Instead, mice treated with the inhibitor survived until the scheduled endpoint and showed ameliorated visible signs of the disease. As shown in Figure 4D and 4E, treatment of tumor-bearing Apc^Min/+^ mice with ONX 0914 led to a highly significant decrease in colonic tumor number compared to analysis of tumor number in vehicle treated Apc^Min/+^ mice which developed multiple large tumors in the distal and proximal parts of the large intestine. Additionally, we observed that spleen hypertrophy was reduced after treatment with the LMP7 inhibitor but not in samples from vehicle treated Apc^Min/+^ mice, as shown in Figure 4F and 4G. Collectively, we conclude that ONX 0914 treatment greatly suppressed clinical signs of the disease and tumor progression in already adenoma-bearing Apc^Min/+^ mice.

### Genetic deficiency of the LMP7 subunit ameliorates spontaneous formation of intestinal polyposis in Apc^Min/+^ mice

**Figure 5:**
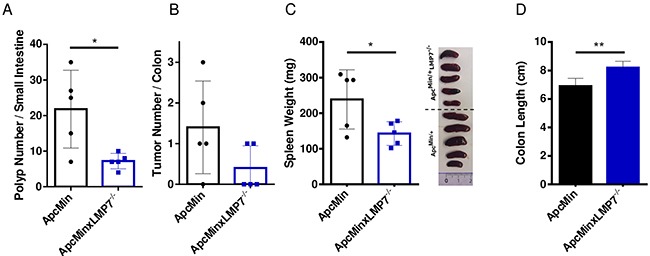
Reduced intestinal tumor burden in Apc^Min/+^LMP7^-/-^ mice 16-week old Apc^Min/+^ (black dots) or Apc^Min/+^LMP7^-/-^ (blue squares) mice were analyzed for colonic tumor formation and small intestinal polyps (n = 5). **A.** The whole intestine was removed and opened longitudinally for macroscopic assessment of tumor and polyp formation. Number of small intestinal polyps of Apc^Min/+^ or Apc^Min/+^LMP7^-/-^ mice are shown; data are means ± SEM. **B.** Graph shows colonic tumor number, as defined by lesions ≥ 2 mm. Data are means ± SEM. **C.** Spleen weight of Apc^Min/+^ or Apc^Min/+^LMP7^-/-^ mice were assessed. According pictures of individual spleens are shown on the right. The graph represents data as means ± SEM. **D.** Average colon length of Apc^Min/+^ (black column) or Apc^Min/+^LMP7^-/-^ (blue column) mice at the age of 16 weeks. *p < 0.05, **p < 0.005.

As it is unknown whether loss of immunopro-teasome subunits interferes with intestinal tumorigenesis in an inflammation-independent tumor environment setting, we investigated the role of LMP7 in intestinal tumor formation upon loss of Apc by breeding Apc^Min/+^LMP7^-/-^ mice. We examined intestinal polyposis of 112 day-old Apc^Min/+^ mice, when intestinal polyps are already well-developed. The total tumor number in the small intestines and colons of Apc^Min/+^ wild type mice correlated with average tumor numbers observed in other studies [36]. Despite the well-known severe intestinal phenotype observed in 16-weeks old Apc^Min/+^ mice, crossing LMP7 onto the Apc^Min/+^ background intriguingly exhibited a significant reduction of tumor lesions particularly in the small intestine (Figure 5A). Macroscopic analysis of colonic tumor lesions showed variable infrequent tumor numbers in wild-type Apc^Min/+^ mice. However, there was a trend towards reduced or even suppressed tumor formation in the large intestine in Apc^Min/+^LMP7^-/-^ mice as shown in Figure 5B. The average size distribution of colonic and small intestinal lesions was similar in Apc^Min/+^ mice and Apc^Min/+^LMP7^-/-^ mice, which may indicate that following loss of Apc, LMP7 deletion impedes tumor initiation rather than tumor growth independent of intestinal inflammation. Nevertheless, LMP7 deficiency ameliorated indirect parameters of disease progression in Apc^Min/+^ mice. Signs of anemia were reduced, since only wild-type Apc^Min/+^ mice showed pale feet and discoloration of internal organs by 16 weeks of age. We also noted that Apc^Min/+^LMP7^-/-^ mice did not develop grossly enlarged spleens compared to spleens isolated from wild type Apc^Min/+^ mice which also weighed significantly more (Figure 5C). Additionally, wild type Apc^Min/+^ mice exhibited decreased colon lengths compared to the Apc^Min/+^LMP7^-/-^ mice (Figure 5D).

In conclusion, these data indicate that the immunoproteasome contributes to the initiation of intestinal carcinogenesis, since deletion of the LMP7 subunit ameliorates spontaneous development of Apc mutation-mediated polyposis in Apc^Min/+^ mice in the absence of experimental exposure to inflammatory agents. Hence, our study identifies the immunoproteasome as a novel candidate drug target in the treatment of familial adenomatous polyposis.

## DISCUSSION

During the past decade, proteasome inhibition has become an established treatment for multiple hematological malignancies such as multiple myeloma or mantel cell lymphoma [37]. Several preclinical studies showed that transformed cells are significantly more sensitive to proteasomal inhibition compared to normal cells [38] as they heavily increase their proteasomal dependency due to increased protein turnover and proliferation in these cells [39]. It has been shown that proteasome inhibitors enhance anti-proliferative, pro-apoptotic, anti-tumor and anti-angiogenic effects. Proteasome inhibitors such as the FDA approved dipeptide boronic acid analogue bortezomib (PS-341, Velcade®) or the irreversible inhibitor carfilzomib (PR-171, Kyprolis®) preferentially induce several apoptotic pathways and inhibition of transcription factors, including NF-κB or c-Myc, in cancer cells while sparing non-malignant cells. Accompanied downregulating of pro-inflammatory cytokines such as IL-6 contributes to inhibition of tumor growth by proteasome inhibitors. Several groups also reported an upregulation of p53 and mediation of cell cycle arrest by increased expression of cdk inhibitors p27 and p21 during proteasome inhibitor-induced anti-myeloma therapeutic treatment [40].

The immunoproteasome has recently gained more recognition as a potential therapeutic drug target to improve current anti-cancer therapies as it is selectively expressed in cells of the immune system and inflamed tissues. At least in mice, immunoproteasome inhibition has a much broader therapeutic window and less adverse side effects than general proteasome inhibition. Cutting edge studies report that immunoproteasomes regulate pivotal pathways that are involved in tumor development and progression including apoptosis and induction of inflammatory responses. Selective inhibition of the immunoproteasome was shown to block growth of leukemic cell lines and patient-derived primary cells by triggering apoptosis. Additionally, selective inhibition of LMP2 by compound UK-101 has been shown to act anti-tumorigenic in a prostatic cancer cell line [41]. However, bortezomib as well as combination therapies with irinotecan were not effective in CRC patients [42]. Recently, our group described an important involvement of the immunoproteasome in cytokine-mediated inflammation in mice as inhibition of LMP7 alters differentiation into inflammatory effector cells and the release of pro-inflammatory cytokines such as IL-6, which plays a pivotal role in cancer [14, 15, 22].

In the present study, we show that immuno-proteasome inhibition offers great promise for the treatment of CRC. We demonstrate an anti-oncogenic effect of inhibition of the LMP7 subunit using the peptidomimetic epoxyketone ONX 0914 in several mouse models of colorectal cancer. ONX 0914 treatment lead to significantly decreased tumor burden in colitis-associated cancer (Figure 1), as well as in the Apc^Min/+^ mouse model (Figure 3). Although the underlying mechanisms are still unknown, we could clearly demonstrate that ONX 0914 impedes colorectal tumorigenesis in long-term treatment schedules as well as in a therapeutic treatment in the absence of acute inflammatory stimuli (Figures 2 and 4). Furthermore, we identified a protective role of genetic deficiency of LMP7 in spontaneous tumorigenesis upon loss of the APC gene (Figure 5), as well as for the establishment of adenomas and/or adenocarcinomas in inflammation-mediated tumor models (Figures 1, 2, 3). These findings suggest an important role of the chymotrypsin like activity of LMP7 in regulation of colon cancer development. Since also the LMP7 deficient mice are protected from colon tumor formation, this argues that ONX 0914 exerts its effect by directly targeting LMP7 and not due to an ‘off target’ effect. Additionally, it shows that LMP7 has a pivotal function during the pathogenesis of chronic colitis and colon cancer development, which cannot be accomplished by compensatory incorporation of the constitutive subunit β5 into the 20S proteasome. Nevertheless, it should be pointed out that at the concentration of 10 mg/kg ONX 0914 applied to mice in this study, the constitutive 20S proteasome subunit β5 was already partially inhibited in a previous study in blood and kidney [14]. Therefore, we can not exclude that a partial β5 inhibiton also occurred in the intestine in this study and that such an inhibition contributed to the therapeutic effect.

The importance of the immunoproteasome in regulation of colorectal tumorigenesis is further highlighted by the fact that activity of immunoproteasomes is generally up-regulated in cancer cells after being exposed to inflamed tumor microenvironment [43]. Additionally, inflamed mouse and human tissue show increased expression levels of immunoproteasome subunits probably contributed by the release of TNF or IFNγ from infiltrating lymphocytes and monocytes already constitutively expressing immunoproteasomes. A high expression of immunoproteasomes was also documented in the uninflamed and inflamed large and small bowel [44–46].

In order to exclude solely anti-inflammatory aspects of the inhibitor, which are already well-described in colitis models [22], we analyzed whether ONX 0914 modulates the anti-tumor response by blocking the tumor promoting character in a therapeutic setting of both mouse models used. Healing after DSS administration is known to enable nearly complete recovery to normal intestinal integrity within 4-5 days, which is in concert with our starting point of treatment as seen in Figures 2A and 4A. ONX 0914 treatment inhibited progression of tumor growth but also suppressed further development of intestinal colonic polyposis (Figures 2D and 4E). These data contains supporting evidence that LMP7 inhibition also plays a protective role in the tumor microenvironment of already established tumors in the absence of overt inflammatory conditions. ONX 0914 may assist the endogenous response to resolve residual pro-inflammatory and pro-cancerogenous responses, thereby limiting colorectal cancer formation. Although underlying mechanisms of treatment efficacy of immunoproteasome inhibition still need to be elucidated, we surmise the anti-inflammatory action of ONX 0914 as the main potential to combat CRC, since all tumors are characterized by an inflammatory tumor microenvironment even if tumor development is not subsequent to chronic inflammation [4]. Inflammation appears to be one of the major mechanisms associated with the development of colorectal tumorigenesis and cancer progression [47], underlined by the fact that chronic inflammation is supposed to be the driving force for development of IBD-associated CRC [48]. Compared to CAC, where inflammation proceeds CRC development, most sporadic CRC malignancies trigger an intrinsic inflammatory response which builds up upon the pro-tumorigenic microenvironment [49]. Both, adenoma-carcinoma mediated and inflammation-associated tumorigenesis, are characterized by inflammatory dysregulation, leading to intestinal adenoma formation. Experimental models of inflammation-associated colon carcinogenesis as well as murine models with germline Apc mutations frequently show massive infiltration of inflammatory cells as well as elevated levels of cell-derived pro-inflammatory cytokines and growth factors directly promoting colorectal tumor cell proliferation, survival, and angiogenesis [9, 50, 51]. Tumor-specific cytokines, such as IL-6, TNF, as well as Th17-related cytokines like IL-17A, IL-21 or IL-23, are highly upregulated in the intestinal tumor microenvironment of human and experimental mouse models and play important roles in pro-inflammatory responses which contributes to formation of a tumor-supportive microenvironment and enhances inflammation-related tumor progression [7, 31].

We show that ONX 0914 treatment reduces IL-6 levels in the early acute colitis phase as well as in later stages of tumor progression (Figure 1K). Decreased IL-6 secretion might correlate with CRC regression or blockage of tumor formation, as the change of the cytokine profile in the colon even at early time points might create the formation of an anti-tumorigenic microenvironment thus restricting the colorectal adenoma-carcinoma development. Of particular note, IL-6 is known as a direct promotor of cancer cell proliferation, induction of angiogenesis, and metastasis in inflammation-associated carcinogenesis and sporadic CRC. The cytokine-driven AOM/DSS model showed increased levels IL-6, mainly produced by lamina propria myeloid cells to promote proliferation and survival of premalignant intestinal epithelial cells, thus enhancing initiation and progression of CAC [52]. Likewise human CRC exhibits increased IL-6 levels in both serum and tumor biopsies and has been also found to positively correlate with tumor load in colon cancer patients. The tumor promoting effect of IL-6 is mainly exerted by activation of the oncogenic transcription factors STAT3 and NF-κB [9, 10], whereas loss of SOCS3 is important for further CAC progression [53]. We hypothesize, that the therapeutic effect of the LMP7 inhibitor is presented with downregulation of cytokines which control the immune and inflammatory milieu to enhance tumor progression in CAC.

Additionally, it has been reported that polyp formation is accompanied by increased inflammation in Apc^Min/+^ mice despite the initiation of adenoma development by mutation of the tumor suppressor gene [51, 54]. Thus, we hypothesize that targeting IL-6 by ONX 0914, as the main contributor to colitis-associated as well as sporadic CRC, might reduce intestinal inflammation and therefore reduce the subsequent risk for colon cancer formation. Downregulation of inflammation is further verified by histology, since ONX 0914 treated colon sections exhibit reduced inflammatory infiltrates and less advanced pathological grading of the tumor microenvironment (Figures 1I and 3G), thereby suppressing colon tumorigenesis. We detected increased numbers of lymphoid follicles in distal colons of vehicle treated Apc^Min/+^ mice (Figure 3G). The increase of lymphoid tissue was shown to correlate with the development of gross tumor lesions [55].

Furthermore, we also observed decreased secretion of IL-17A in ONX 0914 treated colon tissue of AOM/DSS mice compared to vehicle control (Figure 1K). Increased expression levels of IL-17A in blood and cancer tissues have been shown to be associated with the development of CAC [7]. IL-17A was shown to promote angiogenesis by upregulation of pro-angiogenic factors secreted from tumor cells, subsequently promoting carcinogenesis [56]. CRCs with a Th17-dependent immune profile always have poor prognosis in human patients [6]. Inflammation-associated mouse models of colon cancer show that IL-17A^-/-^ mice exhibit milder sign of colitis and tumor growth and reduced levels of the pro-inflammatory cytokines IL-6 and TNF [8]. Thus, immunoproteasome inhibitors would be effective in the therapy of CRC due to their suppressive impact on Th17 responses and the production of IL-23 or IL-6 by myeloid cells. Since IL-6 is an important cytokine in promoting Th17 development [57], we expect that reduced IL-6 levels in ONX 0914 treated mice also reduces pro-inflammatory Th17 cell population and their related cytokines such as IL-17A.

Further evidence for the anti-inflammatory potential of ONX 0914 treatment also in the Apc^Min/+^ mouse model is provided by the phenotype of restored normal spleen sizes after ONX 0914 treatment. Splenomegaly develops due to polyposis-associated inflammation that has become systemic, in agreement with elevated levels of pro-inflammatory cytokines in the serum. Alongside with splenomegaly formation, Chae *et al*. [7] observed thymic atrophy in Apc^Min/+^ mice. Ablation of IL-17A corrected both of these immune abnormalities suggesting that T cell mediated responses contribute to suppression of spontaneous intestinal tumorigenesis. Thus, ONX 0914 treatment as well as LMP7 deficiency probably influence IL-17A cytokine levels in Apc^Min/+^ mice, thus suppressing tumor growth. Although, we did not yet analyze the cytokine and chemokine expression profile of the tumor microenvironment in the Apc^Min/+^ mouse models, Baltgalvis *et al*. [52] showed the importance of Th17-related cytokines in the regulation of tumorigenesis in Apc^Min/+^ mice. Inhibition of IL-17A and the Th17 stabilizing cytokine IL-23 inhibited intestinal hyperplasia and tumor formation in Apc^Min/+^ mice arguing in favor of a cytokine-dependent mechanism of this carcinogenesis model [31]. Moreover, TNF and IL-6 directly control and contribute to tumor promotion in early lesions of Apc^Min/+^ mice by inhibition of apoptosis and up-regulation of angiogenic mediators. Thus, even in the absence of chronic inflammation, CRCs exhibit immune inflammatory infiltrates known as ‘tumor-elicited inflammation’ [4]. We hypothesize, that ONX 0914 treatment also downregulations pro-inflammatory cytokine secretion thus inhibiting tumor formation in Apc^Min/+^ mouse models.

We showed higher apoptotic activity in the tumor-adjacent areas after ONX 0914 treatment (Figure 1J), which implies that ONX 0914 increases apoptotic rates also in the tumor microenvironment to destroy preneoplastic lesions and further promote the tumor-suppressive environment. However, in order to determine a direct effect of ONX 0914 treatment on the tumor cell compartment, it would be necessary to further analyze increased signs of apoptosis also in tumor areas of early and advanced lesions compared to tumor microenvironment as shown in Figure 1J. The immunoproteasome inhibitor may have direct inhibitory effects on cell growth via disturbed protein degradation in malignant colon epithelial cells and the accumulation of poly-ubiquitinated proteins, which is known to induce apoptosis and proliferative arrest in these cells. Besides, ONX 0914 may exert its suppressive and anti-tumor effect on inflammation promoting cells such as neutrophils or M1 macrophages, which appear to be pivotal contributors to the development and progression of adenomatous polyps [58]. Tumor-associated macrophages (TAMs) infiltrating the colon cancer tissue play indispensable roles in inflammation and carcinoma development by directly promoting tumor growth, angiogenesis and metastasis [59, 60].

Although further investigations of key signaling components and other drivers of the initiation or progression of colon cancer are warranted to understand the specific mode of the observed anti-tumorigenic action of ONX 0914, this study shows that inhibition of the LMP7 subunit suppresses the pathogenesis of CRC in preventive and therapeutic preclinical models. Since further immunoproteasome inhibitors have lately been developed [61] and since the first immunoproteasome inhibitor is already tested in a clinical trial, such drugs may not only be of interest to ameliorate autoimmune diseases of the gut and other organs but - based on our preclinical data - would deserve to be clinically explored as potential therapeutics against CRC.

## MATERIALS AND METHODS

### Animals and ethics statement

C57BL/6 mice (H-2^b^) were obtained from Charles River (Sulzfeld, Germany). LMP7^-/-^ mice were kindly provided by Dr. John J. Monaco (Department of Molecular Genetics, Cincinnati Medical Center, OH). C57Bl/6J-Apc^Min/+[CD44]^ mice on a pure C57BL/6 background were obtained from Prof. Jan Paul Medema (University of Amsterdam, The Nederlands). LMP7^-/-^ mice were bred with Apc^Min/+^ mice to obtain Apc^Min/+^ LMP7^-/-^ mice. Animals were housed in an air-conditioned, accredited animal facility on a 12 hour light/dark cycle and fed standard, autoclaved laboratory animal diet and water *ad libitum*. Genotypes of mice were verified via PCR using genomic DNA extracted from tail biopsies to determine knockout and Apc heterozygous state. Investigations have been conducted in accordance with ethical standards, the Declaration of Helsinki, German and EU guidelines and have been approved by the animal experimentation review board of Regierungspräsidium Freiburg.

### Immunoproteasome inhibition *in vivo*

ONX 0914 was generously provided by Dr. Christopher J. Kirk (Onyx Pharmaceuticals) and formulated in an aqueous solution of 10% (w/v) sulfobutylether-ß-cyclodextrin and 10mM sodium citrate (pH 6). 10 mg/kg of the inhibitor was administered subcutaneously in a volume of 100 μl or with 100 μl vehicle as control.

### AOM/DSS tumor model

The establishment of the AOM/DSS mouse model was conducted as previously described [62]. CAC was induced in 8 weeks old female C57BL/6 mice by a single intraperitoneal injection of azoxymethane (AOM, 12 mg/kg) (Sigma-Aldrich) and subsequent oral administration of 2 % dextran sulfate sodium salt (DSS) (MW 36,000-50,0000Da, MP Biomedicals) in drinking water *ad libitum* for 5 consecutive days in 3 intermittent cycles of DSS interrupted by 2-week periods without DSS challenge. Mice were sacrificed 8 weeks after the first day of the first DSS cycle. A total of ≥ 10 mice per time point and treatment were tested across 3 independent experiments. Mice were treated with vehicle or 10 mg/kg ONX 0914 s.c., every second day as indicated in the figure legends and the results section. Mice were monitored every other day for clinical signs of illness including rectal bleeding, rectal prolapse, reduced activity and body weight loss. Upon sacrifice at indicated time points, intestines were excised and flushed with PBS. Afterwards, colons were opened longitudinally and the presence of gross colon lesions was evaluated macroscopically. Number and size of tumors was measured with a caliper to evaluate intestinal tumor development. Colonic tumors with a size of ≥ 2 mm in diameter were counted to obtain overall numbers of lesions per group. Tumors, adjacent normal tissue, as well as serum samples were collected and stored at -80°C until required to perform western blotting or ELISA. Colons were then further processed for histopathological analysis.

### Apc^Min/+^ mice and the Apc^Min/+^-DSS model

Apc^Min/+^ mice of different sex with a starting age of 5 weeks were administered orally with 2 % DSS in the drinking water for 7 consecutive days in order to accelerate tumor initiation, as previously reported [34]. Following sacrifice, the small intestine and the colon were resected, opened longitudinally and macroscopically examined as described above. The small intestine was divided by length into three equal sections (proximal, middle, and distal). Polyps on the intestinal segments were counted and their sizes were measured with a caliper. Subsequently, a quantity of colonic and small intestinal lesions were resected and stored at -80 °C until required. Additionally, spleen weight and size was measured.

### Histology

Mouse colons and small intestinal segments were Swiss-rolled and fixed in 10 % formalin followed by paraffin embedding. Tissue sections of 4 μm were stained with hematoxylin and eosin for pathological evaluation using standard procedures. Histopathologic analysis of neoplastic lesions and degree of dysplasia were assessed according to standard criteria and classification of adenomas of the colon. Tumor multiplicity and intestinal pathology, as well as histology scores were determined microscopically using the Leica light microscope DM-RB and ImageQuant LAS 4000 software. In brief, intestinal inflammation and histopathological assessment was assessed as previously defined by Horino *et al*. [63] on the basis of 4 different parameters: [inflammation severity (0-3) + extent of inflammation (0-3) + Crypt damage (0-4)] x % involvement (0-4).

### Immunoblotting

Colon tissue fractions were lysed in RIPA buffer (50 mM TrisHCl pH 7.5, 150 mM NaCl, 1 % NP-40, 0.5 % SDS) supplemented with 2 mM Na_3_VO_4_, 10 mM NaF, 1 mM PMSF and complete protease inhibitor mixture (Roche Pharmaceuticals) on ice. Total protein was quantified using Pierce BCA Protein Assay Kit (Thermo Scientific, Germany). Equal amounts of protein lysates were resolved in reducing conditions on 15% SDS-polyacrylamide gels for electrophoresis and blotted onto PVDF membranes (Millipore, Merck). Blots were incubated using primary antibody against mouse Cleaved Caspase-3 (1:1000; Cell Signaling) followed by secondary HRP-conjugated anti-rabbit Ig antibody (Dako) at a 1:3000 dilution. To ascertain equivalent loading of the lanes, γ-tubulin (1:10000; Sigma) was used as a control. Bands were detected by chemiluminscence using Super Signal West Chemiluminscent Substrate (Thermo Fisher) according to the manufacturer's instructions.

### Analysis of cytokine expression in serum or intestinal tissue

Lysed intestinal tissue or serum samples were analyzed for mouse cytokines by ELISA according to the manufacturer's instructions (eBioscience) and as previously described.

### Statistical analysis

For animal models, statistical significance of comparisons between ONX 0914 and vehicle treated mice was determined by applying two-tailed Student's t-test with P values p < 0.05 scored as statistically significant. For all other analyses, unless noted in figure legends, data from at least three experiments were presented as means ± SEM and analyzed by applying Student's t-test, one-way or two-way ANOVA, where appropriate with p < 0.05 as statistically significant. For ANOVA, we used Bonferroni *post hoc* analysis to compare groups. The Kaplan-Meier method was used to estimate survival distribution between the groups and log-rank tests were applied to compare survival rates. Statistics were performed using GraphPad software (version 6).

## Abbreviations

Apc: adenomatous polyposis coli
AOM: azoxymethane
CAC: colitis-associated carcinogenesis
CRC: colorectal carcinoma
DSS: dextran sodium sulfate
FAP: familial adenomatous polyposis
IBD: inflammatory bowel disease
IFNγ: interferon-gamma
IL: interleukin
i.p.: intraperitoneal
KO: knockout
LMP: low molecular weight protein
Min: multiple intestinal neoplasms
s.c.: subcutaneous
TNF: tumor-necrosis factor.

## References

[1] Ferlay J, Soerjomataram I, Dikshit R, Eser S, Mathers C, Rebelo M, Parkin DM, Forman D, Bray F (2015). Cancer incidence and mortality worldwide: sources, methods and major patterns in GLOBOCAN 2012. Int J Cancer.

[2] Fearon ER (2011). Molecular genetics of colorectal cancer. Annu Rev Pathol.

[3] Ullman TA, Itzkowitz SH (2011). Intestinal inflammation and cancer. Gastroenterology.

[4] Grivennikov SI, Greten FR, Karin M (2011). Immunity, inflammation, and cancer. Cell.

[5] Galon J, Costes A, Sanchez-Cabo F, Kirilovsky A, Mlecnik B, Lagorce-Pagès C, Tosolini M, Camus M, Berger A, Wind P, Zinzindohoué F, Bruneval P, Cugnenc PH (2006). Type, density, and location of immune cells within human colorectal tumors predict clinical outcome. Science.

[6] Tosolini M, Kirilovsky A, Mlecnik B, Fredriksen T, Mauger S, Bindea G, Berger A, Bruneval P, Fridman WH, Pagès F, Galon J (2011). Clinical impact of different classes of infiltrating T cytotoxic and helper cells (Th1, Th2, Treg, Th17) in patients with colorectal cancer. Cancer Res.

[7] Chae WJ, Gibson TF, Zelterman D, Hao L, Henegariu O, Bothwell AL (2010). Ablation of IL-17A abrogates progression of spontaneous intestinal tumorigenesis. Proc Natl Acad Sci U S A.

[8] De Simone V, Pallone F, Monteleone G, Stolfi C (2013). Role of TH17 cytokines in the control of colorectal cancer. Oncoimmunology.

[9] Grivennikov S, Karin E, Terzic J, Mucida D, Yu GY, Vallabhapurapu S, Scheller J, Rose-John S, Cheroutre H, Eckmann L, Karin M (2009). IL-6 and Stat3 are required for survival of intestinal epithelial cells and development of colitis-associated cancer. Cancer Cell.

[10] Bollrath J, Phesse TJ, von Burstin VA, Putoczki T, Bennecke M, Bateman T, Nebelsiek T, Lundgren-May T, Ö Canli, Schwitalla S, Matthews V, Schmid RM, Kirchner T (2009). gp130-mediated stat3 activation in enterocytes regulates cell survival and cell-cycle progression during colitis-associated tumorigenesis. Cancer Cell.

[11] Wang K, Grivennikov SI, Karin M (2013). Implications of anti-cytokine therapy in colorectal cancer and autoimmune diseases. Ann Rheum Dis.

[12] Rock KL, Goldberg AL (1999). Degradation of cell proteins and the generation of MHC classI-presented peptides. Annu Rev Immunol.

[13] Groll M, Ditzel L, Löwe J, Stock D, Bochtler M, Bartunik HD, Huber R (1997). Structure of 20S proteasome from yeast at 2.4 A resolution. Nature.

[14] Muchamuel T, Basler M, Aujay MA, Suzuki E, Khalid W, Lauer C, Sylvain C, Ring ER, Shields J, Shwonek P, Parlati F, Demo SD, Bennett MK (2009). A selective inhibitor of the immunoproteasome subunit LMP7 blocks cytokine production and attenuates progression of experimental arthritis. Nat Med.

[15] Groettrup M, Kirk CJ, Basler M (2010). Proteasomes in immune cells: more than peptide producers?. Nat Rev Immunol.

[16] Kniepert A, Groettrup M (2014). The unique functions of tissue-specific proteasomes. Trends Biochem Sci.

[17] Qureshi N, Vogel SN, van CI Wayes, Papasian CJ, Qureshi AA, Morrison DC (2005). The proteasome: a central regulator of inflammation and macrophage function. Immunol Res.

[18] Ichikawa HT, Conley T, Muchamuel T, Jiang J, Lee S, Owen T, Barnard J, Nevarez S, Goldman BI, Kirk CJ, Looney RJ, Anolik JH (2012). Beneficial effect of novel proteasome inhibitors in murine lupus via dual inhibition of type i interferon and autoantibody-secreting cells. Arthritis Rheum.

[19] Nagayama Y, Nakahara M, Shimamura M, Horie I, Arima K, Abiru N (2012). Prophylactic and therapeutic efficacies of a selective inhibitor of the immunoproteasome for Hashimoto's thyroiditis, but not for Graves’ hyperthyroidism, in mice. Clin Exp Immunol.

[20] Basler M, Mundt S, Muchamuel T, Moll C, Jiang J, Groettrup M, Kirk CJ (2014). Inhibition of the immunoproteasome ameliorates experimental autoimmune encephalomyelitis. EMBO Mol Med.

[21] Kalim KW, Basler M, Kirk CJ, Groettrup M (2012). Immunoproteasome subunit LMP7 deficiency and inhibition suppresses Th1 and Th17 but enhances regulatory T cell differentiation. J Immunol.

[22] Basler M, Dajee M, Moll C, Groettrup M, Kirk CJ (2010). Prevention of experimental colitis by a selective inhibitor of the immunoproteasome. J Immunol.

[23] Schmidt N, Gonzalez E, Visekruna A, Kuhl AA, Loddenkemper C, Mollenkopf H, Kaufmann SH, Steinhoff U, Joeris T (2010). Targeting the proteasome: partial inhibition of the proteasome by bortezomib or deletion of the immunosubunit LMP7 attenuates experimental colitis. Gut.

[24] Park JE, Ao L, Miller Z, Kim K, Wu Y, Jang ER, Lee EY, Kim KB, Lee W (2013). PSMB9 codon 60 polymorphisms have no impact on the activity of the immunoproteasome catalytic subunit B1i expressed in multiple types of solid cancer. PLoS One.

[25] Imanishi T, Kamigaki T, Nakamura T, Hayashi S, Yasuda T, Kawasaki K, Takase S, Ajiki T, Kuroda Y (2006). Correlation between expression of major histocompatibility complex class I and that of antigen presenting machineries in carcinoma cell lines of the pancreas, biliary tract and colon. Kobe J Med Sci.

[26] De Robertis M, Massi E, Poeta ML, Carotti S, Morini S, Cecchetelli L, Signori E, Fazio VM (2011). The AOM/DSS murine model for the study of colon carcinogenesis: from pathways to diagnosis and therapy studies. J Carcinog.

[27] Boivin GP, Washington K, Yang K, Ward JM, Pretlow TP, Russell R, Besselsen DG, Godfrey VL, Doetschman T, Dove WF, Pitot HC, Halberg RB, Itzkowltz SH (2003). Pathology of mouse models of intestinal cancer: consensus report and recommendations. Gastroenterology.

[28] Karim BO, Huso DL (2013). Mouse models for colorectal cancer. Am J Cancer Res.

[29] Fodde R, Brabletz T (2007). Wnt/beta-catenin signaling in cancer stemness and malignant behavior. Curr Opin Cell Biol.

[30] Su LK, Kinzler KW, Vogelstein B, Preisinger AC, Moser AR, Luongo C, Gould KA, Dove WF (1992). Multiple intestinal neoplasia caused by a mutation in the murine homolog of the APC gene. Science.

[31] Grivennikov SI, Wang K, Mucida D, Stewart CA, Schnabl B, Jauch D, Taniguchi K, Yu GY, Osterreicher CH, Hung KE, Datz C, Feng Y, Fearon ER (2012). Adenoma-linked barrier defects and microbial products drive IL-23/IL-17-mediated tumour growth. Nature.

[32] Potter JD (1999). Colorectal cancer: molecules and populations. J Natl Cancer Inst.

[33] Shoemaker AR, Gould KA, Luongo C, Moser AR, Dove WF (1997). Studies of neoplasia in the Min mouse. Biochim Biophys Acta.

[34] Tanaka T, Kohno H, Suzuki R, Hata K, Sugie S, Niho N, Sakano K, Takahashi M, Wakabayashi K (2006). Dextran sodium sulfate strongly promotes colorectal carcinogenesis in Apc(Min/+) mice: inflammatory stimuli by dextran sodium sulfate results in development of multiple colonic neoplasms. Int J Cancer.

[35] You S, Ohmori M, Peña MM, Nassri B, Quiton J, Al-Assad ZA, Liu L, Wood PA, Berger SH, Liu Z, Wyatt MD, Price RL, Berger FG (2006). Developmental abnormalities in multiple proliferative tissues of Apc Min/+ mice. Int J Exp Pathol.

[36] McCart AE, Vickaryous NK, Silver A (2008). Apc mice: models, modifiers and mutants. Pathol Res Pract.

[37] Ruschak AM, Slassi M, Kay LE, Schimmer AD (2011). Novel proteasome inhibitors to overcome bortezomib resistance. J Natl Cancer Inst.

[38] Ma MH, Yang HH, Parker K, Manyak S, Friedman JM, Altamirano C, Wu ZQ, Borad MJ, Frantzen M, Roussos E, Neeser J, Mikail A, Adams J (2003). The proteasome inhibitor PS-341 markedly enhances sensitivity of multiple myeloma tumor cells to chemotherapeutic agents. Clin Cancer Res.

[39] Adams J (2004). The proteasome: a suitable antineoplastic target. Nat Rev Cancer.

[40] Bogner C, Ringshausen I, Schneller F, Fend F, Quintanilla-Martinez L, Häcker G, Goetze K, Oostendorp R, Peschel C, Decker T (2003). Inhibition of the proteasome induces cell cycle arrest and apoptosis in mantle cell lymphoma cells. Br J Haematol.

[41] Wehenkel M, Ban JO, Ho YK, Carmony KC, Hong JT, Kim KB (2012). A selective inhibitor of the immunoproteasome subunit LMP2 induces apoptosis in PC-3 cells and suppresses tumour growth in nude mice. Br J Cancer.

[42] Kozuch PS, Rocha-Lima CM, Dragovich T, Hochster H, O’Neil BH, Atiq OT, Pipas JM, Ryan DP, Lenz HJ (2008). Bortezomib with or without irinotecan in relapsed or refractory colorectal cancer: results from a randomized phase II study. J Clin Oncol.

[43] Vigneron N, Van den Eynde BJ (2012). Proteasome subtypes and the processing of tumor antigens: increasing antigenic diversity. Curr Opin Immunol.

[44] Visekruna A, Slavova N, Dullat S, Gröne J, Kroesen AJ, Ritz JP, Buhr HJ, Steinhoff U (2009). Expression of catalytic proteasome subunits in the gut of patients with Crohn's disease. Int J Colorectal Dis.

[45] Kremer M, Henn A, Kolb C, Basler M, Moebius J, Guillaume B, Leist M, Van den Eynde BJ, Groettrup M (2010). Reduced immunoproteasome formation and accumulation of immunoproteasomal precursors in the brains of lymphocytic choriomeningitis virus-infected mice. J Immunol.

[46] Vigneron N, Van den Eynde BJ (2014). Proteasome subtypes and regulators in the processing of antigenic peptides presented by class I molecules of the major histocompatibility complex. Biomolecules.

[47] Oshima H, Oshima M (2012). The inflammatory network in the gastrointestinal tumor microenvironment: lessons from mouse models. J Gastroenterol.

[48] Waldner MJ, Neurath MF (2009). Colitis-associated cancer: the role of T cells in tumor development. Semin Immunopathol.

[49] Mantovani A, Allavena P, Sica A, Balkwill F (2008). Cancer-related inflammation. Nature.

[50] Rizzo A, Pallone F, Monteleone G, Fantini MC (2011). Intestinal inflammation and colorectal cancer: a doubleedged sword?. World J Gastroenterol.

[51] Wang L, Zhang Q (2015). Application of the Apc(Min/+) mouse model for studying inflammation-associated intestinal tumor. Biomed Pharmacother.

[52] Baltgalvis KA, Berger FG, Pena MM, Davis JM, Muga SJ, Carson JA (2008). Interleukin-6 and cachexia in ApcMin/+ mice. Am J Physiol Regul Integr Comp Physiol.

[53] Li Y, de Haar C, Chen M, Deuring J, Gerrits MM, Smits R, Xia B, Kuipers EJ, van der Woude CJ (2010). Disease-related expression of the IL6/STAT3/SOCS3 signalling pathway in ulcerative colitis and ulcerative colitis-related carcinogenesis. Gut.

[54] McClellan JL, Davis JM, Steiner JL, Day SD, Steck SE, Carmichael MD, Murphy EA (2012). Intestinal inflammatory cytokine response in relation to tumorigenesis in the Apc(Min/+) mouse. Cytokine.

[55] Cameron IL, Garza J, Hardman WE (1996). Distribution of lymphoid nodules, aberrant crypt foci and tumours in the colon of carcinogen-treated rats. Br J Cancer.

[56] Liu J, Duan Y, Cheng X, Chen X, Xie W, Long H, Lin Z, Zhu B (2011). IL-17 is associated with poor prognosis and promotes angiogenesis via stimulating VEGF production of cancer cells in colorectal carcinoma. Biochem Biophys Res Commun.

[57] Mangan PR, Harrington LE, O’Quinn DB, Helms WS, Bullard DC, Elson CO, Hatton RD, Wahl SM, Schoeb TR, Weaver CT (2006). Transforming growth factor-beta induces development of the T(H)17 lineage. Nature.

[58] Gounaris E, Erdman SE, Restaino C, Gurish MF, Friend DS, Gounari F, Lee DM, Zhang G, Glickman JN, Shin K, Rao VP, Poutahidis T, Weissleder R (2007). Mast cells are an essential hematopoietic component for polyp development. Proc Natl Acad Sci U S A.

[59] Condeelis J, Pollard JW (2006). Macrophages: obligate partners for tumor cell migration, invasion, and metastasis. Cell.

[60] De Palma M, Lewis CE (2013). Macrophage regulation of tumor responses to anticancer therapies. Cancer Cell.

[61] Kisselev AF, Groettrup M (2014). Subunit specific inhibitors of proteasomes and their potential for immunomodulation. Curr Opin Chem Biol.

[62] Popivanova BK, Kostadinova FI, Furuichi K, Shamekh MM, Kondo T, Wada T, Egashira K, Mukaida N (2009). Blockade of a chemokine, CCL2, reduces chronic colitis-associated carcinogenesis in mice. Cancer Res.

[63] Horino J, Fujimoto M, Terabe F, Serada S, Takahashi T, Soma Y, Tanaka K, Chinen T, Yoshimura A, Nomura S, Kawase I, Hayashi N, Kishimoto T, Naka T (2008). Suppressor of cytokine signaling-1 ameliorates dextran sulfate sodium-induced colitis in mice. Int Immunol.

